# Targeting visual-sensory and cognitive impairments following lateral ankle sprains: a practical framework for functional assessment across the return-to-sport continuum—Part 1. Sensory reweighting and cognitive impairments: what are we really talking about and why clinicians should consider central alterations in return to sport criteria

**DOI:** 10.3389/fspor.2025.1668224

**Published:** 2025-10-23

**Authors:** Brice Picot, Alexandre Maricot, François Fourchet, Alli Gokeler, Bruno Tassignon, Ronny Lopes, Alexandre Hardy

**Affiliations:** ^1^ Inter-University Laboratory of Human Movement Biology (LIBM), Savoie Mont-Blanc University, Chambéry, France; ^2^ French Society of Sports Physical Therapy (SFMKS Lab), Pierrefitte-sur-Seine, France; ^3^ Human Physiology and Sports Physiotherapy Research Group, Faculty of Physical Education and Physiotherapy, Vrije Universiteit Brussel, Brussels, Belgium; ^4^ Department of Physiotherapy, La Tour Hospital, Meyrin, Switzerland; ^5^ Exercise Science and Neuroscience, Department of Exercise & Health, Paderborn University, Paderborn, Germany; ^6^ Faculty of Health, Amsterdam University of Applied Science, Amsterdam, Netherlands; ^7^ REVAL Rehabilitation Research Center, Faculty of Rehabilitation Sciences, Hasselt University, Diepenbeek, Belgium; ^8^ Centre Orthopédique Santy, Hôpital Privé Jean Mermoz, Lyon, France; ^9^ Clinique du Sport, Paris, France

**Keywords:** lateral ankle sprains, chronic instability, cognition, sensory reweighting, return to sport

## Abstract

Lateral ankle sprain (LAS) is the most common traumatic injury, with a high recurrence rate and chronic ankle instability (CAI) developing in ∼40% of cases. LAS leads to patho-mechanical, sensory-perceptual and motor-behavioral deficits. Poor management of the return-to-sport (RTS) is now considered a major cause of re-injury and development of CAI, particularly due to the lack of validated tests and the failure of existing ones to account for those central deficits. The first part of this topic aimed to clarify concepts of cognitive constructs and sensory reweighting and their association with CAI. We also aimed to identify objective RTS criteria and discuss their limits regarding their ability to encompass central impairments. Motor-cognitive deficits have been identified using computerized cognitive tasks and dual-task paradigms. More specifically, deficits in visual memory, processing speed or inhibitory control and attentional resource allocation have demonstrated reduced performance in CAI populations. In addition, altered sensory reweighting process towards visual input has also been observed. While objective criteria are crucial to prevent re-injury, current evaluations remain largely subjective and central impairments are unaccounted for in conventional RTS testing. The Ankle-GO^TM^ score was recently developed to guide clinicians in decision making process. To date, it is the first validated score that could help to identify patients who will RTS at the same level, those at risk of recurrence and those who are more likely to become copers. Unfortunately, it does not target cognitive or sensory reweighting alterations, that are both relevant in sport to manage gameplay demands.

## 1 Introduction

Lateral Ankle Sprain (LAS) is the most common injury in sports (1), and up to 40% of patients will develop chronic ankle instability (CAI) (2). This condition is characterized by a history of LAS resulting in a feeling of ankle instability, episodes of giving way and/or recurrent sprains as well as loss of function reported during daily activities and sports (3, 4). Ultimately, LAS has substantial consequences on patients, ranging from socio-economic impact to a diminished quality of life, often associated with the early onset of ankle osteoarthritis (5).

CAI is characterised by a spectrum of symptoms related to the ankle itself. According to the integrative model proposed by Hertel and Corbett (4), pathomechanical impairments represent a key component contributing to the development and perpetuation of chronic ankle instability (CAI). These impairments encompass structural and mechanical alterations that disrupt normal joint function. Notably, recurrent ankle sprains may lead to ligamentous laxity, altered arthrokinematics, and insufficient passive restraint, which in turn compromise joint congruency and load distribution. Additionally, deficits in dorsiflexion range of motion and postural alignment changes can modify movement patterns and increase stress on adjacent structures. Together, these mechanical disruptions establish a maladaptive foundation that predisposes individuals to persistent symptoms and recurrent injury.

Other factors that are not physically identifiable during routine examinations may also be present. Hertel and Corbett (4) emphasize that sensory-perceptual impairments play a central role in the CAI continuum. These deficits primarily reflect disrupted afferent input from peripheral mechanoreceptors following LAS. Diminished somatosensory feedback, particularly from the ligaments and surrounding soft tissues, can alter joint position sense and impair proprioception (6). Such changes compromise the central nervous system's ability to accurately perceive limb orientation and movement, thereby reducing sensorimotor control. As a result, individuals with CAI often exhibit delayed or inappropriate neuromuscular responses during dynamic tasks, further increasing the risk of reinjury and perpetuating functional limitations.

The same authors (4) highlight these motor-behavioral impairments as a critical component influencing long-term functional outcomes. They refer to maladaptive changes in motor planning and execution that emerge as a consequence of repeated injury and altered sensory input. During dynamic tasks, CAI patients often develop compensatory movement strategies, such as reduced joint excursions, increased co-contraction, or altered muscle recruitment patterns (7, 8). These protective behaviors are driven by fear of reinjury or reduced confidence in the ankle's stability. Altered motor pattern becomes progressively ingrained, contributing to performance deficits and perpetuating the cycle of instability.

This establishes the concept of a neurosignature unique to each patient, functioning as a form of individual and multifactorial identity profile (4). This interaction is particularly relevant because it enables us to interact with our environment accordingly. The brain sits between this interaction and influences this feedback loop constantly and brain neuroplasticity is frequently observed following LAS (9). The set of brain changes associated with CAI (=a neurosignature involving structural and functional adaptations) may affect not only how individuals respond to external stimuli, but also higher-order cognitive processes such as attention, working memory, and inhibition (10). Lastly, recent evidence suggests alterations in the sensory reweighting process (11–13), with an increased visual reliance among patients suffering from CAI (14, 15). One of the main reasons for the burden of LAS, particularly the high recurrence rate and the development of CAI, is poor management of the return-to-sport (RTS) (16). Conventional RTS assessments do not take these recent data into account and therefore do not target the central deficits that may be present among patients.

The overall objective of this two-part article (mini-review and perspective) is to summarize current knowledge on the central deficits associated with CAI, as well as existing RTS criteria. We will also propose a new tool for assessing cognitive deficits and sensory reweighting alteration in CAI patients to assist clinicians in their decision-making process, based on recent data.

In the first part, we present a synthesis of scientific literature addressing cognitive impairments and alterations in sensory reweighting associated with CAI. We also summarize the current literature regarding objective RTS criteria and discuss their limitations. Furthermore, we outline potential approaches for improving the assessment of central deficits through the implementation of dual-task paradigms.

In the second part (Targeting Visual-Sensory and Cognitive Impairments Following Lateral Ankle Sprains: A Practical Framework for Functional Assessment Across the Return-to-Sport Continuum. Part 2: From theory to practice: recommendations for optimizing Return To Sport after lateral ankle sprains using cognitive and visual-sensory assessments, Frontiers in Sports and Active Living, [under review]), we propose a β “brain” extension of the Ankle-GO™ score, which currently represents the only objective RTS criterion. This extension integrates dual-task conditions into each functional item of the score, in order to capture potential cognitive and sensory reweighting deficits in patients. In addition, we describe a framework for quantifying cognitive cost under dual-task conditions (DTC), thereby enabling clinicians to more effectively interpret the outcomes of this β “brain” extension.

## 2 Understanding central mechanisms and their implications in dynamic tasks

Too often, the field of cognition and its implications in dynamic tasks are haphazardly conflated or even confused—with topics related to sensory/visual reweighting. Given their potential importance in the context of LAS, we will clearly define what cognition and sensory reweighting process precisely are in the next paragraphs.

Human motor control emerges from an integrated network of cortical and subcortical regions. The primary motor cortex executes voluntary actions, while the premotor and supplementary motor areas coordinate planning and sequencing (17, 18). Higher-order cognitive control is provided by the prefrontal cortex, while the basal ganglia and cerebellum regulate movement initiation, learning, and fine-tuning (19, 20). Sensory integration is mediated by the posterior parietal cortex, which combines visual and proprioceptive information, with the cerebellum and vestibular pathways (17, 21). Then the superior parietal and association cortex further weight feedback to enhance motor control (13).

### 2.1 Cognition

According to Diamond (22), cognition is the study of cognitive processes or functioning in connection with the particular neural mechanisms that underlie them in the brain and any impairment of these mechanisms. In many sports situations that require focus, coordination, and control to override internal or external stimuli, higher-level cognitive functions, also known as “executive functions”, are crucial. As a collection of adaptive behaviors that enable athletes to successfully navigate the environment by shifting and adapting to changing environmental cues and needs, executive functions are defined as the capacity to coordinate cognitive, emotional, and motor processes (22). It is possible to distinguish between three primary executive functions: cognitive flexibility, working memory, and inhibition. The ability to regulate one's thoughts, behavior, attention, and/or emotions to overcome a strong internal inclination to act or an outside distraction is known as inhibition, or inhibitory control (22). Working memory, which describes a person's capacity to retain and hold information in an active, readily retrievable state while blocking out distractions and interference, is closely related to inhibitory control (22, 23). Cognitive flexibility is the ability to modify cognitive processing techniques in response to novel situations (22). For instance, processing speed (such as reaction time), visual attention, and dual tasking are examples of lower-level cognitive abilities. Information processing speed is the rate at which an athlete processes new information and the amount of time needed to retrieve previously stored information from memory. Information processing speed is a fundamental cognitive function required for more complex functions like working memory. It characterizes an athlete's capacity to perceive, process, and react to a sensory stimulus. A common metric for evaluating an athlete's ability to react quickly to a given stimulus is reaction time. The attempt to complete two or more tasks at the same time is known as dual tasking or multitasking (24). It is believed that training cognitive functions can be used to enhance one or more facets of sports performance by better understanding the distinct cognitive functions that underpin sports performance, both domain-general and domain-specific (25). This approach has been criticized, though, because it might not be sport-specific given the complexity of athletic settings (24). While domain-specific cognitive skill training is thought to have a higher transfer to sports performance because of its higher ecological validity, domain-general cognitive skill training is argued to not necessarily transfer to sports performance (25, 26).

### 2.2 Sensory reweighting

Sensory reweighting refers to the central nervous system's (CNS) ability to dynamically adjust the relative importance (or “weight”) of different sensory inputs (i.e visual, vestibular, and somatosensory/proprioceptive) to maintain balance and posture (27–29). This process allows individuals to adapt to changing environmental conditions in order to maintain optimal postural control. The relative weight of each sensory system depends on factors such as task complexity, environmental conditions, and the accuracy of sensory input. For example, when standing on firm surfaces, the CNS primarily relies on proprioception and vision to maintain balance. However, when standing on an unstable surface, the CNS reduces reliance on somatosensory input and shifts to more reliable sources, such as visual cues if available. In eyes-closed (EC) conditions, an increased reliance on somatosensory cues is observed (21).

There are different ways to identify visual contribution during postural control. Recently, the development of strobe glasses allows patients to perform dynamic tasks under perturbed vision, whereas full EC conditions only permit static tasks (14, 30). By constraining visual input (i.e stroboscopic vision) during dynamic activities such as hopping or jumping, clinicians can assess sensory reweighting towards vision. A significant decrease in performance under SV conditions, particularly in comparison to the uninjured limb or healthy individuals, may indicate increased reliance on visual input.

To summarize, successful performance in dynamic tasks relies on the integrated function of both lower- and higher-order cognitive processes. Lower-order cognition involves the fundamental, often automatic, processing of sensory input, while higher-order cognition encompasses the complex mechanisms of attention, memory, decision-making, and executive control (31). Together, these processes shape how information is interpreted and acted upon by integrating incoming sensory data with prior knowledge and task goals to guide behavior. In parallel, sensory reweighting is an adaptive process where the central nervous system dynamically shifts its reliance among sensory modalities (e.g., vision, proprioception, vestibular) based on environmental conditions and task demands. A key distinction is that while cognition governs the interpretation of information and subsequent decision-making, sensory reweighting adjusts the input signals themselves to optimize sensorimotor control.

**Figure 1:**
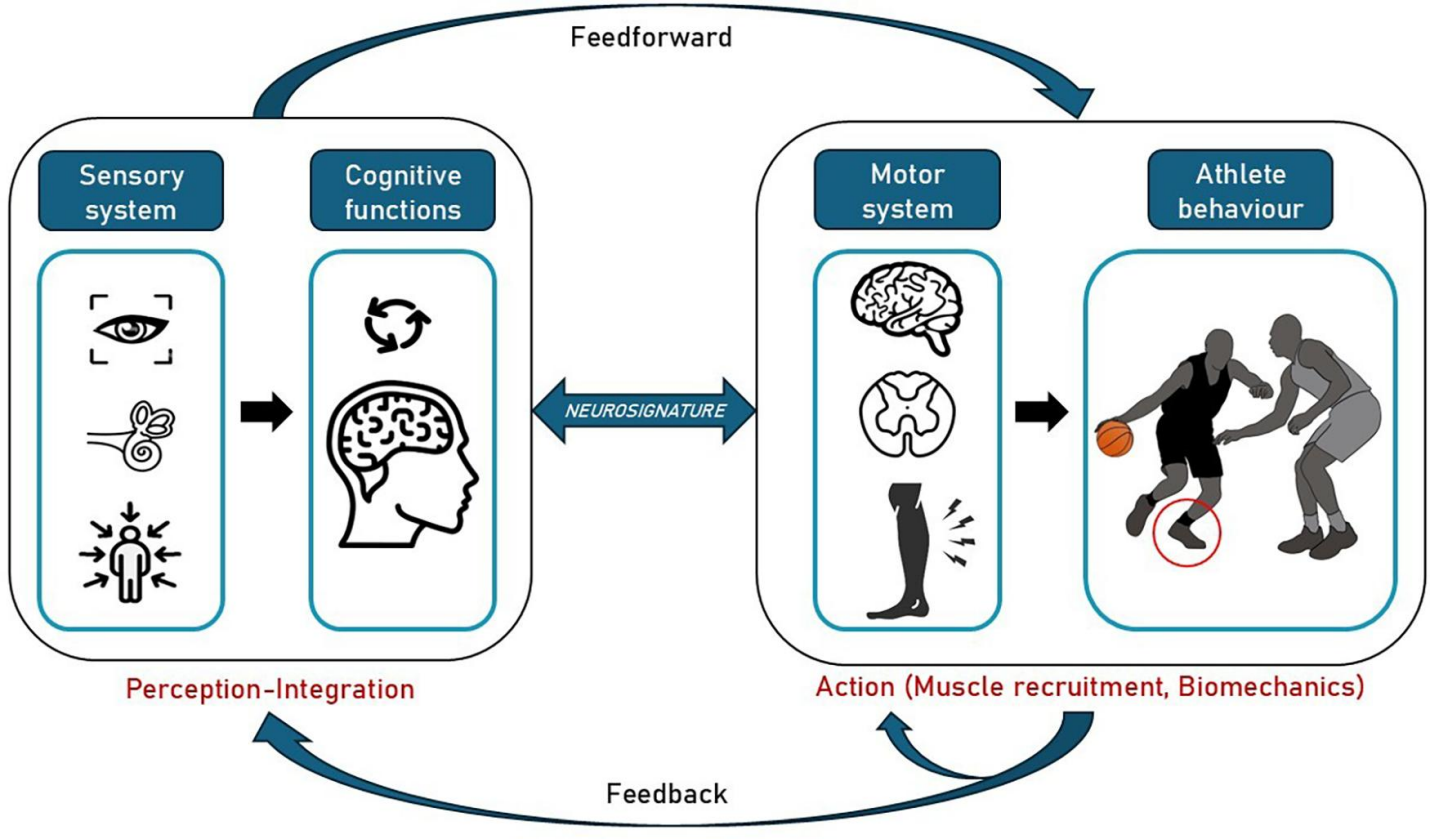
Motor control model driving specific athlete behaviour in a game situation with high risk of lateral ankle sprain. This involves perception of the environment (sensory informations), rapid and effective integration of these cues (cognitive functions), resulting in the production of afferents (motor system) leading to specific athlete biomechanics (action) to the game situation (Neurosignature).

A decline in either cognitive function (affecting interpretation and decision-making) or sensory reweighting abilities (disrupting the quality of sensory input) can reduce motor performance. This creates a potential sensorimotor mismatch, where the brain's commands and the body's feedback are misaligned, increasing the risk of injury (Figure 1). These alterations are commonly described in patients with CAI and may explain the high rate of recurrence in this population.

## 3 Central alterations and RTS management following LAS

In the context of ankle impairments and drawing on Hertel's model (4)—particularly the sensorimotor loop—numerous clinical and practical applications can be identified.

### 3.1 Cognitive functions

Recent studies have identified subtle cognitive impairments in CAI mostly assessed using computerised cognitive tasks (CNT) and dual-task paradigms (10). The most commonly used paradigms to assess cognitive deficits were the number generation or digit span task and the serial subtraction performed during single-leg stance or gait (10).

Seated CNT have revealed deficits in visual memory (32), while evidence for impairments in attention (32–34) and processing speed is mixed (32, 34–38). Dual-task assessments have provided additional evidence for compromised cognitive-motor integration in CAI (39–44). Specifically, dual-task paradigms that tested inhibitory control and attentional resource allocation have demonstrated reduced performance in CAI populations compared to healthy controls (45, 46). This is particularly relevant given the functional overlap between perception–action coupling and executive control: many sport-specific or daily tasks require cognitive regulation of movement, especially when there is time pressure, adaptability is required. Understanding this interplay offers important insight into the central mechanisms that may underpin persistent functional deficits in CAI.

Indeed, CAI patients often exhibit longer reaction times, reduced spatial perception, and impaired memory (32, 38, 42, 47, 48). These deficits result in a reduced ability to respond to a dynamic and unpredictable environment, which is particularly common in sport, thereby increasing the likelihood of sensorimotor mismatch.

It should be noted that all mentioned studies relied relatively small patient samples (*n* < 30) and presented heterogeneity in the inclusion criteria for CAI, which may account for result variability (10, 39). Differences in dual-task outcomes could also reflect task difficulty and heterogeneity within CAI groups. Despite the accumulation of scientific data, the level of evidence remains limited and additional high-quality studies are needed to better understand, identify, and target cognitive deficits in patients with LAS/CAI.

### 3.2 Sensory reweighting

An increased reliance on visual information has been identified in CAI patients compared to healthy individuals during single-leg stance (13–15). While inconsistent results have been observed in eyes-open conditions, CAI patients almost consistently exhibit postural control impairments during eyes-closed conditions. Inadequate sensory reweighting may contribute to the functional deficits observed in CAI, caused by over- or under-reliance (beyond or under utilising what's optimal) on specific types of sensory input. For instance, while increased reliance on visual input may help maintain balance during traditional rehabilitation exercises, this strategy often breaks down in more complex, sport-specific environments. In such setting visual resources are already heavily engaged in managing gameplay demands such as tracking opponents, anticipating ball trajectories, and responding to unpredictable events.

The exact cause of this mechanism among CAI patients remains unclear. Since somatosensory receptors, such as articular receptors or muscle spindles, are frequently disrupted following LAS, loss of proprioception is frequently observed (6, 49). It could be argued that the CNS is enabled to overcome this loss of proprioceptive signals and shift to compensate for its reliance on visual information. Recent results (13) revealed that CAI patients show stronger but less stable functional connectivity between the superior parietal cortex and visual cortices, as well as greater variability in connectivity with the spinocerebellum, which correlates with increased visual reliance. Overall results align with Freeman's articular deafferentation theory (50).

Increased visual reliance is therefore considered as a compensatory mechanism that could partially explain postural control impairments and functional deficits observed following LAS and the high rate of recurrences. Therefore, it is crucial to identify individuals who rely excessively on vision for balance and implement rehabilitation strategies to restore appropriate sensory reweighting following injury.

Overall results confirm that central deficits occurred following LAS and could exist among CAI patients. However, it should be noted that there are significant discrepancies regarding inclusion criteria and the definition of CAI patients across studies. We recommend that authors follow the IAC guidelines when including CAI patients (3). More specifically, patient should (i) have suffered a history of at least one significant ankle sprain at least 12 months prior to the study enrolment, (ii) reported ankle joint “giving way”, and/or recurrent sprain and/or “feelings of instability” on the same ankle and (iii) reported diminished self-reported function.

Poor management of RTS is a key contributor to high recurrence rates and the development of CAI. Given recent data, it therefore seems essential to target central alterations throughout the RTS phase, particularly in sports patients performing dual-task situations. The following section reviews the current management of RTS, existing validated objective criteria and identifies their potential limitations in this regard.

### 3.3 Management of RTS

Return-to-Sport decisions are critical at the end of rehabilitation, particularly in populations such as individuals with CAI, where the risk of re-injury remains high. While physical recovery (e.g., restoration of range of motion, strength, and balance) is necessary, it is not sufficient to ensure safe and sustained RTS (51). A systematic review highlighted the lack of objective criteria to safely guide return-to-sport decisions (52). To date, there is no consensus on the specific criteria to be used, and decisions are still largely based on time-based guidelines (16).

Research shows that nearly half of athletes resume their sports activities the day after the injury, and within a week, up to 80% have returned to play (53, 54). However, most athletes do so without having fully recovered from the impairments caused by the sprain—such as deficits in postural control and joint range of motion (55, 56).

An expert consensus conducted by the International Ankle Consortium (IAC) emphasized the importance of evaluating five key domains before clearing an athlete to return to sport (57). The authors proposed a new “PAASS” framework to evaluate Pain, Ankle-specific impairments, Athlete perception (including kinesiophobia and psychological readiness), Sensorimotor control, and Sport-specific functional performance to guide clinicians in assessing readiness. Unfortunately, it does not specify how clinicians should assess these items.

### 3.4 The Ankle-GO™ score

This tool is a cluster of six items selected on their relevance for monitoring LAS patients (58, 59) and the recommendation of PAASS framework (57). All items and threshold values were selected based on their ability to distinguish between healthy individuals, copers, and patients with CAI. Finally, they were selected if they demonstrated sufficient reliability and validity and did not require specific or expensive equipment. The total duration of the Ankle-GO™ test does not exceed 30 min.

It has been recently developed and validated among patients suffering from CAI and could help to identify patients who will RTS at the same level of play (58), those who will suffer reinjury (60), and those who are more likely to become coper (61). The total score is 25 points (Table 1) spread over two self-reported questionnaires as well as four functional tests.

**Table 1:** Ankle-GO scoring system [adapted from (58)].

Items		Raw scores	Weight	Maximum score
FAAM	Activities of Daily Living	<90%	0	2
		90%–95%	1	
		>95%	2	
	Sport	<80%	0	2
		80%–95%	1	
		>95%	2	
ALR-RSI		<55%	0	3
		55%–63%	1	
		63%–76%	2	
		>76%	3	
SLS		>3 errors	0	3
		1–3 errors	1	
		0 error	2	
		No feeling of instability	+1	
mSEBT (in % of limb length)		COMP <90%	0	7
		COMP 90%–95%	2	
		COMP >95%	4	
		ANT >60%	+1	
		PM >90%	+1	
		No feeling of instability	+1	
SHT		>13 s	0	5
		10–13 s	2	
		<10 s	4	
		No feeling of instability	+1	
F8T		>18 s	0	3
		13–18 s	1	
		<13 s	2	
		No feeling of instability	+1	
Ankle-GO™ Score				25

#### Ankle ligament reconstruction–return to sport after injury (ALR-RSI)

The ALR-RSI questionnaire assesses the psychological readiness of athletes to RTS following an ankle sprain (62–65). It includes 12 items rated from 0 (no confidence) to 10 (full confidence), with the total score converted to a percentage. Based on the original ACL-RSI and adapted for ankle injuries, the tool reflects the athlete's confidence and emotions, a higher score indicating better psychological readiness. LAS patients scoring above 46% two months after injury were more likely to return to preinjury level of sport or higher at 4 months (62).

#### Foot and ankle ability measure (FAAM)

The FAAM is a patient-reported outcome measure composed of two subscales: the Activities of Daily Living (FAAM-ADL, 21 items) and the Sports subscale (FAAM-Sport, 8 items) (66). Each item is rated on a 5-point Likert scale from 0 (unable to perform) to 4 (no difficulty). Scores are converted into percentages for each subscale, providing an overview of functional limitations. The FAAM is validated for CAI (67), higher scores indicate better self-reported function. Cut-off scores of 90% and 80% in ADL and Sports subscales, respectively, are used to identify patients with CAI (3). In addition, individuals are commonly considered copers if they score greater than 95% on both subscales (4).

#### Single-leg stance test (SLS)

The SLS test evaluates static postural control. The subject stands barefoot on one leg, eyes closed, with hands on hips and a slightly flexed knee (10°) for 20 s (68, 69). Examiner reports the number of balance errors during the tests: lifting hands-off iliac crest, opening eyes, stepping, stumbling or falling, moving hip into more than 30° of flexion or abduction, lifting forefoot or heel and remaining out of the test position more than 5 s (Table 1). A low number of errors indicates good static postural control. A proposed 3-error cut-off score is commonly used to identify CAI patients (68).

#### Modified star excursion balance test (mSEBT)

The mSEBT assesses dynamic postural control across three directions: anterior (ANT), posteromedial (PM), and posterolateral (PL) (70, 71). Standing barefoot on one leg, the patient reaches with the other leg in each direction and returns to the initial position without losing balance. The trial is canceled if the subject lifts any part of the stance foot, removes his/her hands from the hips or transfers weight to the other limb. The distance is recorded (in cm) and evaluated in relation to the limb length (from the anterior and superior iliac spine to the medial malleolus). A composite score (COMP) is calculated as the average of the three directions. After 4 learning trials in each direction for each leg, 3 trials are recorded and averaged (Table 1). Lower reaching distance indicates poorer dynamic postural control. Individuals scoring below 94% and 89.1% in the COMP score are more likely to get injured, and a cut-off score of 91% in the PM direction is described to identify CAI patients (68, 72, 73).

#### Side hop test (SHT)

The SHT evaluates lateral agility and neuromuscular control (74, 75). The patient hops side-to-side across two lines spaced 30 cm apart, completing 10 cycles as quickly as possible. The first hop is directed outward. Only valid hops (i.e without touching the lines) are counted. Completion time is recorded (Table 1). A cut-off score of 12.9 s has been calculated to identify CAI patients (68), with values below 10 s observed in the uninjured limb or healthy patients (74).

#### Figure-of-8 test (F8T)

The F8T is an agility test where the patient hops on one limb in a figure of 8 pattern as fast as possible between two cones 5 meters apart. The patient has to perform two consecutive laps, for a total distance of 20 m) (74). The time taken to complete the exercise is recorded, with a longer time reflecting poorer single-limb hopping performance. A cut-off score of 17.4 s was calculated to identify CAI patients (68), with values below 12 s observed in the uninjured limb or healthy patients (74).

An additional point is awarded for each of the four functional tests (SLS, mSEBT, SHT, F8T) if the subject does not report any feeling of instability during the activity (Table 1). This subjective measure accounts for perceived stability of the ankle, which is a key factor in chronic ankle instability (74).

Recent results revealed that following LAS, patients who score below 8 pts on the Ankle-GO™ score are less likely to RTS at the same level of play and 9 times more likely to suffer a reinjury within 2 two years (58, 60). In addition, those who score above 11 pts are 12 times more likely to become LAS copers (61). Lastly, after lateral ankle reconstruction for CAI patients, a cut-off score of 6 points allows to identify those who will return to sports (odd ratio = 18) (76).

The Ankle-GO™ score demonstrates good construct validity and internal consistency (Cronbach's α of 0.79) and excellent test–retest reliability (ICC = 0.99, with a minimal detectable change of 1.2 points), but these findings are based on a relatively small and homogeneous sample (64 LAS patients and 30 controls) (58). The tool was validated exclusively in physically active patients, limiting generalizability to elite athletes, adolescents, and older adults. Moreover, although discriminant and predictive validity were supported with Area under the Receiver Operating Characteristic Curve (AUC) = 0.77 for predicting RTS at 4 months, AUC = 0.75 for predicting reinjury over 2 years and AUC = 0.7 for predicting copers, these figures fall in the “fair to good” range, indicating that this score cannot be used as a standalone test for RTS.

## 4 Discussion

Despite very promising results, the Ankle-GO™ score is not perfect, particularly because it does not encompass dual-task situations or visual constraints that could highlight central deficits in patients. Yet, emerging theories suggest that central factors also play a pivotal role in ensuring a safe RTS, particularly in sports requiring cognitive constraints with dual tasks situations where visual attention is dedicated to the management of a ball or an opponent (Figure 1). Unfortunately, only few RTS evaluations involved cognitive constraints (47, 77). Thus, there is an urgent need to develop functional performance tests that incorporate cognitive and visual perturbations in patients with CAI (10, 32, 78, 79).

For example, during a hopping test, it is possible to add a secondary (cognitive) task such as counting backwards, memorizing and repeating a sequence of numbers, or reacting to a stimulus (color, sound, word). Performance can be evaluated by comparing the results of the motor test alone (distance, stability, contact time…) with those obtained under dual-task conditions. In parallel, cognitive errors and reaction time can be analyzed. Any deterioration in performance or cognition may indicate which task is being prioritized by the patient. This approach makes it possible to assess an individual's ability to maintain motor control while attention is divided, which more closely reflects the demands of real sporting activities. Yet, the impact of motor-cognitive interference can be quantified using Dual-Task Cost (DTC) (80).

$$\mathrm{DTC}=\frac{\mathrm{Dual}\;\;\mathrm{task}\;\mathrm{performance}\;\text{-}\;\mathrm{Single}\;\mathrm{task}\;\mathrm{performance}\;}{\mathrm{Single}\;\;\mathrm{task}\;\mathrm{performance}}\;\times 100$$

Separate DTC values for motor and cognitive domains help identify which system is compromised and how task prioritization may influence performance.

Incorporating motor-cognitive testing and analyzing task prioritization strategies during RTS evaluation after LAS provides a more ecologically valid measure of functional recovery. These assessments can reveal persistent deficits in neuromotor control or cognitive flexibility that traditional tests miss. Ultimately, a dual-task framework enhances the clinician's ability to make informed, individualized RTS decisions that reduce reinjury risk and support long-term athletic performance. These aspects will be largely discussed in the part 2 of this article (Targeting Visual-Sensory and Cognitive Impairments Following Lateral Ankle Sprains: A Practical Framework for Functional Assessment Across the Return-to-Sport Continuum. Part 2: From theory to practice: recommendations for optimizing Return To Sport after lateral ankle sprains using cognitive and visual-sensory assessments, Frontiers in Sports and Active Living, [under review]).

### 4.1 Clinical implications for rehabilitation

Athletes recovering from CAI often revert to novice-like motor patterns, necessitating the relearning of previously automatic skills. This regression stems from the adoption of maladaptive movement strategies that increase reliance on cognitive resources and visual input, thereby reducing motor efficiency. Due to compromised sensorimotor pathways, these individuals typically engage in more conscious control of movement, which places greater demands on attentional capacity and slows reaction time. Athletes with CAI may exhibit increased attention directed toward their injured ankle, as they consciously monitor movement patterns and joint stability (81–83). This self-attentional focus is further reinforced during rehabilitation, when rehabilitation specialists frequently provide internal focus instructions—directing the athlete's attention to specific body mechanics, such as knee alignment or muscle activation (84). These cues may inadvertently contribute to excessive cognitive load. This adaptation reduces cognitive resources available for other tasks (85, 86). To restore automaticity, an external focus of attention—such as concentrating on the outcome of movement rather than its mechanics—can help free up cognitive resources. This shift enables enhanced cerebellar involvement in sensorimotor control, fostering improved internal modeling for predictive adjustments and real-time motor corrections.

According to Gibson's ecological theory of perception, movement and sensory information are inherently linked in a continuous feedback loop (87). Movement generates sensory information by interacting with the environment, while sensory input, in turn, guides and refines movement. This bidirectional relationship allows individuals to adapt their actions based on real-time environmental cues. In the context of sports, athletes rely on this dynamic interplay to adjust their positioning, timing, and force production in response to rapidly changing game conditions. This interplay between movement and sensory information highlights the need for rehabilitation programs that incorporate enriched environments to facilitate optimal recovery. By exposing patients to practice variability and/or different situational conditions, they can actively explore and refine movement patterns in response to real-time sensory input.

This framework applies particularly to athletic populations of patients with LAS/CAI, especially those who participate in sports involving dual tasks or risky movements, such as jumping, landing, and cutting movement. It also applies to athletes whose vision is focused on managing their environment (movements and positioning of opponents and teammates) or tracking the trajectory of a ball, for example.

A recent meta-analysis reveals that dual-task training may be effective in improving static and dynamic postural stability among CAI patients but confirms the need for more high-quality studies to confirm the short and long-term effectiveness (43). This applies regardless of age, level of practice or severity of injury. It also seems important to introduce dual-task situations and neurocognitive exercises, as well as assessments targeting these elements, with the aim of primary injury prevention among these athletes.

In the second part of this topic (Targeting Visual-Sensory and Cognitive Impairments Following Lateral Ankle Sprains: A Practical Framework for Functional Assessment Across the Return-to-Sport Continuum. Part 2: From theory to practice: recommendations for optimizing Return To Sport after lateral ankle sprains using cognitive and visual-sensory assessments, Frontiers in Sports and Active Living, [under review]), we will propose a “β(rain)” extension of the Ankle-GO™ score that could help clinicians to target sensory-visual and cognitive deficits among patients following LAS in the RTS continuum.

## 5 Conclusion

LAS are not solely peripheral injuries but lead to neuroplastic changes affecting sensory integration and cognition. These central alterations can undermine traditional rehabilitation and RTS decision-making. Unfortunately, objective RTS criteria are lacking and the only predictive tool currently available does not include visual perturbation or cognitive constraints. We encourage the International Ankle Consortium (IAC) to promote the inclusion of tools that assess central function in future consensus statements on decision-making regarding return to sport. Clinicians must adopt a neuromotor and neurocognitive approach to fully restore athletic function and reduce recurrence risk.
